# Workhouse Infirmaries

**Published:** 1898-01-01

**Authors:** 


### The Hospital

A Journal of

**The Medical Sciences and Hospital Administration.**

Edited by Sir Henry Burdett, K.C.B., and Solomon C. Smith, M.D., M.R.C.P.

Vol. XXIII.—No. 588.] [Jan. 1, 1898.

### Workhouse Infirmaries.

The *Times* of Monday last contains a long and interesting article on the changes in the administration of the Poor-Law which have been brought about during the reign of Her Majesty, and among these the improvements in the construction and management of workhouse infirmaries receive, as they deserve, a very prominent place. The labours of the Marchioness of Lothian, of Mrs. de Morgan, of Miss Twining, and of Lady Montagu of Beaulieu are given the recognition which they merit, but it is remarkable that those of the originator of the movement, the late Dr. Anstie, by whom the then existing circumstances of the case were mainly brought to light, should be passed over in silence. A belief that the state of workhouse infirmaries was unsatisfactory was strongly entertained by the late Dr. Wakley, the founder of the *Lancet*, who left as a legacy to his successor in the editorial chair of that journal a suggestion that their condition should be investigated. The suggestion bore fruit in the time of his son, the late Dr. James Wakley, who spoke to Dr. Anstie on the subject, and Dr. Anstie, after a conference with Mr. Hart, drew up a scheme for the production of a series of articles, which took the form of "reports" on the workhouse infirmaries of England and Wales.

About the same time, or soon afterwards, Dr. Ellis, then resident medical officer to the St. Pancras Workhouse Infirmary, and now of Portsmouth, took steps to inform the coroner for the district of every death which occurred in the institution nominally under his control, so that inquests might be held and suitable verdicts returned. At Cheltenham, also, similar steps were taken by the late Dr. Fleischmann; and the public mind generally became deeply moved by the disclosures which were made. Dr. Joseph Rogers, medical officer to the Strand Union, who had for several years stood forward as the defender of the poor under his charge, invited the co-operation of Dr. Anstie and of the late Mr. John Storr in the establishment of the Workhouse Infirmary Reform Association, and sought the aid of several influential philanthropists and politicians, including ladies as well as gentlemen. At the very commencement of the proceedings the three founders were set aside, and Dr. Anstie, when urged to assert himself in the matter, declined to do so, saying that he attached more importance to the results of the movement than to the persons by whom it was to be carried into effect. Notwithstanding the somewhat unpromising commencement, excellent work was done, and the attention, not only of the public, but also of the members of Boards of Guardians, was for the first time strongly directed to the requirements of the sick poor, and to the manner in which those requirements should be provided for.

It cannot be denied, however, that the wave of philanthropy in which the movement had its origin, and from which its first impulse was derived, has to some extent spent its force, and that workhouse nursing, far more than workhouse medical attendance, has been falling more and more behind the standard of general nursing which is now maintained. For this there may be some excuse in the character of the cases to be dealt with, they being in a large proportion chronic, in a large proportion not very susceptible of improvement, and in a large proportion due to the errors or misconduct of the sufferers. Taking them all round, they are disheartening to deal with; and it cannot be any matter of surprise if trained nurses, such as they are at the present day, prefer to devote themselves to more promising material. Still, as the article in the *Times* points out, a great deal may be done to render nursing in workhouses more attractive to those who are engaged in it, and who now often seem desirous to escape from it as soon as they can. In the first place, the nursing strength in a workhouse infirmary is seldom sufficient to allow any individual nurse to act up to the standard of what she has been taught to consider her duty; and next, when there is a trained sister at the head of a ward, she is often subject to the control of a matron who knows nothing about nursing, and who, with the rashness commonly incidental to ignorance, likes to exercise authority in matters which, if not beyond her province, are at least beyond her competence. The average guardian, too, has but imperfectly learned the lesson that saving is not always economy, and thus, with the best intentions, may sometimes hinder where he ought to help. A few months ago, if we are not mistaken, Dr. Lovell Drage wished the present state of workhouse nursing to be inquired into and reported upon by the Royal British Nurses' Association, and we hope that his suggestion to this effect may be accepted and acted upon. The motive force supplied in the sixties seems to be exhausted, and the time has fully come for some new action in a matter of national concern.

